# Evaluation of Mobile Apps Targeted to Parents of Infants in the Neonatal Intensive Care Unit: Systematic App Review

**DOI:** 10.2196/11620

**Published:** 2019-04-15

**Authors:** Brianna Richardson, Justine Dol, Kallen Rutledge, Joelle Monaghan, Adele Orovec, Katie Howie, Talia Boates, Michael Smit, Marsha Campbell-Yeo

**Affiliations:** 1 School of Nursing Dalhousie University Halifax, NS Canada; 2 Faculty of Health Dalhousie University Halifax, NS Canada; 3 Centre for Pediatric Pain Izaak Walton Killam Health Centre Halifax, NS Canada; 4 School of Information Management Dalhousie University Halifax, NS Canada; 5 Division of Neonatal Perinatal Medicine, Department of Pediatrics Faculty of Medicine Dalhousie University Halifax, NS Canada; 6 Division of Neonatal Perinatal Medicine, Department of Pediatrics Faculty of Medicine Izaak Walton Killam Health Centre Halifax, NS Canada

**Keywords:** parenting, intensive care units, neonatal, review, mobile health, mHealth, mobile apps, eHealth, education, nonprofessional, infant, premature

## Abstract

**Background:**

Parents of preterm infants increasingly use their mobile phone to search for health information. In a recent review, websites targeted toward parents with infants in the neonatal intensive care unit (NICU) were found to have poor to moderate quality educational material; however, there is a dearth of literature regarding mobile apps for NICU parents.

**Objective:**

This study aimed to identify and evaluate apps targeting parents of infants in the NICU for quality of information, usability, and credibility.

**Methods:**

We systematically searched the Apple App Store and Google Play using 49 key terms (eg, “preterm infant”) from July 26 to August 18, 2017. English apps targeting NICU parents that cost less than $20 were included. Apps for health care professionals, e-books/magazines, or nonrelevant results were excluded. In total, 3 tools were used for evaluation: Mobile Application Rating Scale (MARS) to measure quality; Patient Education Materials Assessment Tool for Audiovisual Materials (PEMAT-AV) to measure the app’s content usability; and Trust it or Trash It to measure credibility.

**Results:**

The initial search yielded 6579 apps, with 49 apps eligible after title and description screening. In total, 27 apps met the eligibility criteria with 9 apps available in both app stores; of those, the app with the most recent update date was chosen to be included in the analysis. Thus, 18 unique apps were included for final analysis. Using MARS, 7 apps (7/18, 39%) received a good score on overall quality (ie, 4.0 out of 5.0), with none receiving an excellent score. In addition, 8 apps (8/18, 44%) received a PEMAT-AV score between 51% and 75% on the understandability subscale, and 8 apps (8/18, 44%) scored between 76% and 100% on the actionability subscale. Trust It or Trash It deemed 13 apps (13/18, 72%) as *trash* for reasons including no identification of sources or lack of current information, with only 5 (5/18, 28%) deemed trustworthy. Reviewer’s expert evaluation found 16 apps contained content that matched information provided by multiple sources; however, most apps did not meet other objective measurement items to support credibility. When comparing the MARS overall quality and subjective quality scores with trustworthiness of apps, there was no statistically significant difference. A statistically significant difference was found between the 2 MARS quality scores, indicating that, on average, apps were ranked significantly lower on subjective quality compared with overall quality measures.

**Conclusions:**

This evaluation revealed that of the available apps targeting NICU parents, less than half should be considered as acceptable educational material. Over two-thirds of the apps were found to have issues regarding credibility and just over a quarter were considered good quality. The apps currently available for NICU parents are lacking and of concern in terms of quality and credibility.

## Introduction

### Background

More than 1 in 10 babies are born preterm (ie, before 37 weeks gestation) worldwide and are often admitted to the neonatal intensive care unit (NICU) to help support their survival, growth, and development [1]. Parents of these infants requiring NICU care typically perceive this experience to be incredibly stressful, emphasized by the unfamiliar medical environment, appearance or behavior of their newborn, and interruptions in developing their role as a parent [2-7]. Unsurprisingly, parents strongly value being informed about their infant’s condition by the NICU care team; however, their communication needs often go unmet. This is often because of the amount of information, either too much or too little information, and timing that health care providers deliver the said information [8]. As a result, parents strive to regain a sense of control by seeking additional information from external sources, including other health care providers, written educational materials, and the internet [8]. Parents often consult the internet, even before a health care provider, using the search engine Google or social media to search for advice related to their child’s health and well-being [9-11]. More specifically, parents with infants in the NICU have also been found to prefer accessing the internet with their mobile phone when seeking information about their infant’s health, with a trend in younger NICU parents preferring to use mobile apps [8,12,13].

Given the prevalence of mobile phone ownership worldwide, leveraging this medium to disseminate health information provides a great opportunity to better support overall health practices [14-16]. Not surprisingly, there has been a surge in the development of mobile health (mHealth) apps [17]. The use of mHealth apps by new parents has been increasing, often during the perinatal period, with mothers reporting an interest in or use of apps to monitor their health and their family’s well-being [18-21]. Although mHealth apps have great potential, there are currently no formal quality standards that are required when developing these resources; thus, little is known about the quality of mHealth apps [14,22].

Broadly, concerns have been reported related to the quality of general health content on the internet [23] and considering this highly vulnerable population, it is imperative to evaluate the web-based health resources targeted to parents of infants in the NICU to ensure they are accessing current and evidence-informed information. To address this, we recently conducted a systematic review of websites available through Google that were targeted to NICU parents and found websites overall to be moderate to poor in terms of their reliability and quality of information [24]. A recent review evaluated parenting apps [25]; however, there was no emphasis on apps for parents with infants in the NICU. Building on our systematic review of Google, this review sought to evaluate the quality of current apps to provide further insight into how best to meet the informational needs of NICU parents.

### Objectives

The primary objective of this review was to identify and evaluate apps available to parents of infants in the NICU for quality (ie, quality of health information and overall design), usability (ie, clarity and applicability of the health information), and credibility (ie, accuracy and reliability of the content). This study had the following research questions:

1. What is the quality of mobile apps targeted to parents of infants in the NICU?
2. What is the usability of the content within mobile apps targeted to parents of infants in the NICU?
3. Is the information provided by mobile apps targeted to parents of infants in the NICU credible?

In addition, a secondary objective was to explore common databases to determine if any peer-reviewed literature regarding the apps included for full review has been published and if so, what has been reported on them.

## Methods

### Study Design

Although there are some differences from traditional review methods related primarily to the search strategy, this study followed systematic review methodology, adhering to Preferred Reporting Items for Systematic Reviews and Meta-Analyses (PRISMA) standards [26]. A protocol for this review was developed a priori and registered through PROSPERO International prospective register of systematic reviews [27]. This study is consistent with numerous recent reviews of mHealth apps using the same methodological approach [25,28-33].

#### Search Strategy

To identify the appropriate records for this review, the Canadian Apple App Store and Google Play were used as databases to search for mobile apps. In 2017, Apple and Android mobile phones accounted for 99.7% of the new market share [15]. Although not all Android phones access Google Play, it has been rated the leading app store offering approximately 1.6 million apps available for download, with Apple App Store in second place offering 1.5 million apps [34]. Thus, the search was limited to Apple App Store and Google Play, given the substantial number of apps offered by these stores. This is consistent with other studies reviewing mobile apps [35].

To ensure a comprehensive inquiry, this review conducted a systematic search using a 2-step approach developed in collaboration with a librarian (KR). In step 1, we searched both app stores using 21 relevant key terms (eg, “parenting,” “newborn,” “preterm infant,” “preemie,” “premature baby,” “neonatal intensive care unit,” “NICU,” and “neonatology”). This was followed by step 2 where each store was searched again using string keywords by inputting multiple forms of parent (eg, “parent,” “caregiver,” “guardian,” “mother,” and “father”) in combination with the following terms: “neonatal intensive care unit,” “NICU,” “special care nursery,” and “neonatal.” This 2-step approach was utilized because of differing search methods between app stores at the time of this review, with the search algorithm for Apple optimized by using single keyword and the algorithm for Google Play with string keywords [35-37].

#### Selection Criteria

For feasibility, the search was limited to the top 100 apps identified from each search term applied, which is consistent with previous studies reviewing apps [38]. This limitation was applied as searches within the Apple App Store will continually refresh with additional apps of increasingly less relevancy. Apps that met inclusion criteria and were available in both stores were evaluated separately at the screening stage to assess if there were any substantial differences across operating systems. However, to limit redundancy during the final stage of this review, the most recently updated app as identified in the store description was kept for analysis. Snowball searching through recommended apps was conducted; however, no new apps were found that met the criteria that were not identified in the original search.

#### Inclusion Criteria

Although the term parent will be used throughout this review, this term will be considered inclusive of guardians, additional family or individuals that provide care to infants in the NICU. We chose against using the term caregiver as it is often considered more suggestive of a health care provider role within this context. Apps targeting parents related to the NICU experience were eligible for inclusion. To ensure a broad reach of available apps, there were no restrictions on the app’s purpose. For example, apps could be for awareness, education, or tracking growth data, or reducing stress and enhancing coping. The following inclusion criteria was determined a priori: (1) apps targeted to parents of infants in the NICU; (2) available through Apple App Store or Google Play, accessible in Canada; (3) English language; (4) free or paid apps costing less than Can $20 (a consistent cut-off with similar studies as general users are unlikely to spend more than $10 per app [32]); (5) apps available in the following Apple App Store categories: Health & Fitness, Lifestyle, Medical, and Social Networking; and (6) apps available in the following Google Play categories: Communication, Education, Health & Fitness, Lifestyle, Medical, Parenting, and Social.

#### Exclusion Criteria

Apps were excluded if they were (1) general parenting apps, not related to the NICU experience; (2) intended for health care professionals; (3) classified as *e-books* by app store description or reviewers.

### Screening Process and Data Extraction

After removal of duplicates, the title and store descriptions of all apps identified in the initial search were screened by 6 reviewers (BR, JD, KR, AO, JM, and KH) to determine eligibility for full review. For data extraction, 3 independent reviewers (BR, AO, and JM) were trained on how to use the measurement tools to ensure consistency when evaluating the apps. For full review, 1 reviewer was assigned to evaluate Android apps (AO), another for Apple apps (JM), and a third reviewer evaluated all apps (BR) using devices from both operating systems. Any disagreements were resolved by consensus or with a fourth reviewer (JD). Apps that were eligible for full review were downloaded and evaluated using 4 mobile phones, 2 Android (HTC One M7; LG G4) and 2 Apple (iPhone 5s; iPhone 6s). To address the primary objectives of this review, data were extracted using a structured data retrieval form compiled using the following 3 measurement tools:

#### Mobile App Rating Scale

The Mobile App Rating Scale (MARS) tool is designed to gather descriptive data and assess the quality of mHealth apps using an objective and reliable method [39]. This tool evaluates an average overall app quality through 4 core subscales: engagement, functionality, aesthetics, and information quality. The tool also offers 2 optional subscales to support the evaluation: app subjective quality and perceived impact of the app on user knowledge and behaviors. See Textbox 1 for the definition of each core subscale. Each item within the core subscales uses a 5-point scale (1=Inadequate, 2=Poor, 3=Acceptable, 4=Good, and 5=Excellent). Subscale scores can be isolated to determine strengths and limitations of the app under evaluation. Subscale mean scores are then summed and averaged to create an overall mean quality assessment. Mean scores are calculated for each subscale and scores classifications are consistent with item responses (ie, 1=Inadequate, 2=Poor, 3=Acceptable, 4=Good, and 5=Excellent). For this study, we report on each scale individually as well as report a total score.

**Definitions of Mobile App Rating Scale core subscales.**

1. Engagement: “Fun, interesting, customizable, interactive, well-targeted to the audience”
2. Functionality: “App functioning, easy to learn, navigation, flow logic, and gestural design of app”
3. Aesthetics: “Graphic design, overall visual appeal, color scheme, and stylistic consistency”
4. Information Quality: “Contains high quality information from a credible source”

**Example of Patient Education Materials Assessment Tool for Audiovisual Materials items.**

1. Understandability: “The material makes its purpose completely evident.”
2. Actionability: “The material clearly identifies at least one action the user can take.”

#### The Patient Education Materials Assessment Tool for Audiovisual Materials

This tool systematically evaluates content usability through the understandability and actionability of audiovisual patient education materials [40]. The Patient Education Materials Assessment Tool for Audiovisual Materials (PEMAT-AV) assesses if materials are understandable and key messages are clear to a diverse population with varying levels of health literacy. By evaluating actionability, the PEMAT-AV assesses if a diverse population can identify what they can or need to do based on the information provided in the education material. Each item has 3 options for scoring (0=Disagree, 1=Agree, and NA=Not Applicable), and overall scores for each subscale are summed and divided into percentage quartiles, with a potential range of 0% to 100%. See Textbox 2 for an example of items to assess understandability and actionability.

#### Trust It or Trash It

To support assessment of credible and unbiased resources, Trust It or Trash It provides guidance on how to critically evaluate the quality of health information provided in health resources [41]. This tool uses 6 questions to help determine the validity and reliability of the resources: Who wrote the information you are reading? Who provided the facts/Where did the facts come from? Who paid for it? When was it written or updated? How do you know this information pertains to you? Does the information seem reasonable based on what you’ve read or know? Each question has an associated description to explain how to evaluate appropriately to either a *trust* or *trash* the resource. Resources will be considered as *trash* if receiving this option for at least 3 questions from this tool. Trust It or Trash It is a relatively simple tool that will complement the remaining measurement tools and strengthen the rigor of this review.

The secondary objective, to identify the existence of peer-reviewed publications of included apps, was conducted by searching the app name included in the full review in PubMed and Google Scholar. This search was conducted in conjunction with data extraction in Fall 2017.

### Data Analysis

Data analysis was conducted using IBM SPSS 24.0. Descriptive statistics and frequencies were used to summarize the results of the evaluations from each measurement tool. As both the MARS tool and Trust It or Trash It tool evaluated quality, we conducted independent *t* test to compare MARS overall quality scores and subjective quality scores on whether an app was found to be trustworthy, as per the Trust It or Trash It tool. Additionally, a dependent *t* test was conducted to compare between MARS overall quality scores and subjective quality scores.

## Results

### Screening Process

**Figure 1:**
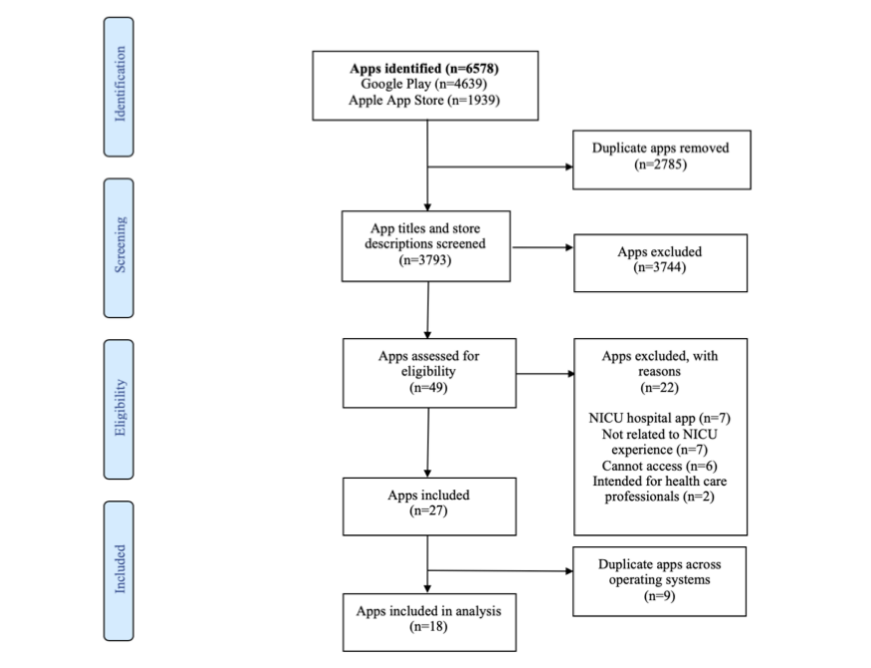
Preferred Reporting Items for Systematic Reviews and Meta-Analyses flowchart of search process. NICU: neonatal intensive care unit.

After systematically searching both stores and manually inputting search findings into a Microsoft Excel spreadsheet over a month in 2017, our initial search yielded 6578 apps. After title and description screening, a total of 49 were assessed for eligibility. A total of 27 apps were included for full review and evaluated by 3 independent reviewers. Moreover, 9 apps were excluded as they were available on both stores; exclusion was based on oldest date as identified in the description, including 6 from Google Play and 3 from Apple App Store. There were 18 unique apps remaining that were included in the final analysis: 6 from Google Play and 12 from the Apple App Store. See Figure 1 for the PRISMA flow diagram outlining the screening process [26].

### Descriptive Characteristics of Apps

Of the 18 unique apps, 89% (16/18) had a last update year of 2015 or later, of which 61% (11/18) were updated in 2017. Although 94% (17/18) of apps were free to download, the 1 app that required payment to download was last updated in 2015. Moreover, 3 apps used commercial advertisements and 1 had in-app purchases, costing users Can $20.99 for a subscription to access more content. The costs of in-app purchases were not identified in the app store description. Although this app was included for full review, only content that was freely accessible was evaluated. Half of the apps were developed in the United States (n=9), with the remaining coming from the United Kingdom (n=3), Australia (n=1), New Zealand (n=1), Canada (n=1), Greece (n=1), or were of unknown origin (n=2). The majority of apps appeared to have been developed by a reputable source including nongovernmental organizations (n=12), government agencies (n=3), or university (n=1). App user rankings are represented by star ratings (5-point scale) in the Apple App Store and Google Play. None of the apps from Apple had an overall star rating, stating that they have not received enough reviews or ratings to rank. Moreover, 4 apps from Google Play had enough user ratings to provide an overall score, ranging from 3 to 5 stars with a median of 4 stars. The number of users who rated the apps ranged from 1 to 127. Table 1 provides a complete list of the included apps and their characteristics (for full table, see Multimedia Appendix 1). Using theoretical strategies predefined by the MARS tool, the purpose of the apps was primarily for providing information or education (n=16), advice or tips (n=12), or monitoring or tracking data (n=10), as shown in Table 2. Although the apps covered a wide range of topics related to the NICU experience, those most commonly addressed were breastfeeding or feeding (n=18), growth and development (n=14), and illness or health issues (n=13). See Table 3 for a list of the topics identified in the apps.

**Table 1:** Description of apps.

App name, operating system	Developer	Country	Version	Cost (Can $)	Ads/in-app cost
Babble, Apple iOS	Midcentral District Health Board	New Zealand	1.5	Free	No
Baby Growth Tracker, Android	St. Rose Dominican Hospital	United States	1.1	Free	No
Connect2NICU^a^, Apple iOS	Connect2 NICU	Unknown	1.1	Free	No
Gift of Life, Apple iOS	Mark Hoewing	United States	1	Free	Yes (Ads)
Integrated Family Delivered Care, Android	Propeller Apps	United Kingdom	1	Free	No
Life’s Little Love, Apple iOS	AlexiaTek	Canada	1.0.8	Free	No
myChildren’s, Android	Nationwide Children’s Hospital	United States	4.2.1.1459-b6334bd	Free	No
MyPreemie App, Apple iOS	Graham’s Foundation	United States	1.13	Free	No
My Neonatal Journal, Apple iOS	Rancon	United Kingdom	1.1.1	Free	No
NICU Companion, Apple iOS	Indiana University	United States	2	Free	No
NICU Parent, Apple iOS	PSD Apps	Greece	1.6	Free	Yes (Ads)
Our Journey in the NICU, Apple iOS	Phoenix Children’s Hospital	United States	1	Free	No
Pebbles of Hope, Apple iOS	Pebbles of Hope	United States	2	Free	No
Peekaboo ICU Preemie, Android	Jozo Radman	United States	0.0.5	Free	No
Premature Baby Journal, Android	Life’s Little Treasures Foundation	Australia	1.1	$2.96	No
Premature Birth, Android	Health Care Tips	Unknown	1	Free	Yes (Ads)
Quantum Caring for Parents (QCP)—NICU, Apple iOS	Caring Essentials	United States	1.1	Free	Yes (in-app purchases)
Tommy’s—My Premature Baby, Apple iOS	Tommy’s	United Kingdom	1.0.5	Free	No

^a^NICU: neonatal intensive care unit.

**Table 2:** Theoretical strategies classified by the Mobile Application Rating Scale (N=18).

Theoretical strategies	Apps, n (%)
Information or education	16 (88)
Advice or tips or strategies or skills training	12 (75)
Monitoring or tracking	10 (55)
Assessment	1 (5)
Goal setting	1 (5)
Peer support	1 (5)

**Table 3:** Full list of topics covered (N=18).

Topic	Statistics, n (%)
Breastfeeding or feeding	18 (100)
Growth and development	14 (77)
Illness or health issues	13 (72)
Overview or expectations	11 (61)
Skin-to-skin care or kangaroo care	11 (61)
Support	10 (55)
Physical health	10 (55)
Experience in the NICU^a^	10 (55)
Bringing baby home	7 (38)
Emotional needs of parents	7 (38)
Long-term outcomes	7 (38)
Complications or risks in the NICU	7 (38)
Parenting or bonding	5 (27)
Pain	2 (11)
Death or loss	2 (11)
Labor or birth	2 (11)
Depression	2 (11)
Preterm birth prevention	1 (5)
Relationships	1 (5)
Entertainment	1 (5)
Parent engagement in care	1 (5)

^a^NICU: neonatal intensive care unit.

### Quality Assessment Using Mobile Application Rating Scale

Using the MARS tool, the average overall quality MARS score of the 18 apps ranged from 2.33 to 4.31, with an average of 3.37 (median 3.37). Less than half of apps (7/18, 39%) received an acceptable score (range: 3.26-3.72) on overall quality, with 28% (5/18) receiving a good score (range: 4.06-4.31), and no apps receiving an excellent score. Overall, apps scored low on engagement (1.00-4.6, mean=2.68) and moderate on functionality (2.75-5.00, mean=3.93), aesthetics (1.3-4.67, mean=3.21), and information quality (2.75-4.50, mean=3.65). See Table 4 for results across each core subscales and overall quality. Interestingly, the 5 apps to receive a good score in aesthetics were the only apps to receive a good score in overall quality. Within the engagement subscale, app’s capabilities for customization were evaluated with 50% of apps having no (n=7) or insufficient options customization (n=2) and the other 50% of apps having basic (n=5) or numerous (n=4) options for customization. The subjective quality of apps varied, with only 11% (2/18) of apps receiving a good score. As part of the subjective quality assessment, evaluators determined 72% (n=13) of apps would likely be used 3 to 10 times (n=6), 1 to 2 times (n=4), or not used at all (n=3) in the next 12 months. Apps varied in their perceived impact on parental knowledge, attitudes, and behavior, with 22% (4/18) of apps receiving an acceptable score, 11% (2/18) receiving a good score, and 6% (1/18) receiving an excellent score. See Multimedia Appendix 1 for the individual app results across each subscale and overall app quality. Quantum Caring for Parents received the highest overall quality score (mean=4.31), followed by MyPreemie App (4.16), NICU Companion (4.10), Babble (4.08), and Integrated Family Delivered Care (IFDC; 4.06).

### Patient Education Materials Assessment Tool for Audiovisual Materials Evaluation

In terms of usability reflected by health literacy, using the PEMAT-AV, 44% (8/18) of the apps received a score between 51% and 75% on understandability and 44% (8/18) were within 76% to 100% on actionability. One app that was solely for tracking and monitoring data was removed from this analysis as the app contained no educational content. See Table 5 for PEMAT-AV results by quartiles.

**Table 4:** Mobile App Rating Scale average scores.

Core subscales	Apps by average score, n				
	Inadequate	Poor	Acceptable	Good	Excellent
Engagement	2	7	8	1	0
Functionality	0	1	7	8	2
Aesthetics	2	3	8	5	0
Information quality	0	3	6	9	0
Overall app quality	0	6	7	5	0

**Table 5:** Patient education material assessment tool audiovisual scores.

Categories	Apps by Patient Education Materials Assessment Tool for Audiovisual Materials score^a^, n			
	<25%	26%-50%	51%-75%	76%-100%
Understandability	1	4	8	4
Actionability	1	5	3	8

^a^Tracking or monitoring only apps were excluded from analysis (n=1).

### Trust It or Trash It

The credibility of apps appears lacking, with 72% (13/18) of apps receiving a *trash* score and only 28% (5/18) deemed as trustworthy. Over 80% (15/18) of apps received a trash score on the following questions: “Who provided the facts or Where did the facts come from?” and “When was it written or updated?” However, with the question “Does the information seem reasonable based on what you’ve read or know?,” nearly all apps (n=16) were considered to match the information found in multiple sources based on the reviewer’s expert evaluation.

### Overall Highest Ranking

The app to rank the highest across all measurement outcomes was IFDC. We conducted a subanalysis comparing quality measures between the MARS tool and Trust It or Trash It tool. There was no statistically significant difference in overall quality of apps reported in the MARS between the apps that were deemed trustworthy (mean 3.65, SD 0.75) compared with those that were not (mean 3.26, SD 0.57) in the Trust It or Trash It tool, *t*_16_=−1.20, *P*=.25. Similarly, no statistically significant difference in the subjective quality of apps was found between apps that were trustworthy (mean 2.85, SD 1.22) compared with those that were not (mean 2.33, SD 0.89), *t*_16_=−1.01, *P*=.33. When comparing within the MARS quality scores of apps, on average apps had a higher overall quality score (mean 3.37, SD 0.63) than the subjective quality score (mean 2.47, SD 0.98), *t*_17_=−7.05, *P*<.001.

### Secondary Objective

Only 1 app, MyPreemie App, had a relevant publication identified through a search in both PubMed and Google Scholar. The paper on MyPreemie App, published in 2013, was a description article that did not report any outcomes on feasibility, uptake, or impact on parent or newborn outcomes [42]. An abstract discussing MyChildren’s app was retrieved, but it provided no mention of the NICU-specific content [43].

## Discussion

### Principal Findings

Parents in the NICU are utilizing the internet and mobile apps easily accessible to them through their mobile phones to specifically search for more information about their infant’s health and well-being [12]. To the authors’ knowledge, this is the first and only review of apps targeting parents of infants in the NICU. Consistent with our previous work that found websites targeted to parents with infants in the NICU to be of moderate to low quality[24], this review found the apps currently available for the same population also lacking in quality and credibility. Overall, just over a quarter of apps were considered good quality, less than half acceptable in terms of educational materials, and over two-thirds as having issues related to credibility.

Over a third of the reviewed apps provided information on key topics regarding the NICU experience including breastfeeding or feeding, growth and development, and illness or health issues. However, information on topics that have been identified as prevalent concerns among NICU parents, such as attachment [44], infant pain [45], and death or loss [46,47], were found within only a small number of apps. Unsurprisingly, the topics covered and the proportion of apps related to these topics was comparable with our previous review of websites [24]. Although topics most commonly covered in the apps align with the increasing emphasis on family-centered or family-integrated care in the NICU, there are still gaps in addressing what parents identify as important. Many apps were updated in 2017, the same year when the review was conducted, and the “What’s New” statements in the app store descriptions included mainly technical aspects, such as fixing delays, as app version updates only occur when there are changes to the software code. Thus, it was difficult to determine if the content was updated within the apps, especially as most apps did not disclose their sources.

### Quality

Similar findings have been identified from recent systematic reviews of various health-related apps [25,29,32,33] predominantly in terms of quality and credibility. Among other studies using the MARS tool, engagement scores of mHealth apps have been consistently low across reviews of mindfulness-based apps [32], cardiovascular disease symptom monitoring and self-management apps [29], and asthma self-management apps [28]. The focus of these reviews suggests that these apps should be inherently engaging to ensure target users believe they are valuable resources to improve their health and well-being. Additionally, our review found only half of apps had little to no options for customization limiting the user’s ability to engage and personalize the app. Using the MARS subjective quality subscale, our expert reviewers evaluated that on average the majority of apps included in this review would likely be used only 0 to 10 times within the next year by NICU parents, which may not be sufficient or valuable for supporting parents throughout their NICU experience. However, this is in contrast to findings that reported almost one hundred percent of NICU parents use the internet or their mobile phones daily [12]. Again, consistent with our evaluation, these reviews found apps to have high scores for their functionality, yet moderate in terms of aesthetics, information quality, and overall quality [28,29,32] This trend suggests that future apps should strive to improve engagement, aesthetics, and information quality to strengthen the overall quality of apps. In addition, a recent review of apps targeting adults with chronic lung disease specifically reported that none of the apps included in their evaluation provided source identification [33]. Although important across all mHealth apps, it is of particular importance for parents with infants in the NICU to have timely access to credible, yet also engaging and understandable information as they often experience feelings of vulnerability and stress during this highly sensitive period [8,48,49].

Of the apps reviewed, most apps received at least an acceptable score in overall quality using the MARS tool. Although the Quantum Caring for Parents app had the highest overall quality MARS score, this app requires a paid subscription (ie, Can $20.99) to access all of the content, which may not be feasible for all parents. Although the other highest-ranking apps were free, it is important to note how the financial support model for these free apps (eg, grant funding) could impact quality over time if the developers are not able to sustain the app. Although outside of the scope of this review, it would be interesting to explore the relationship between app quality and financial support. Only 3 apps received a high score on perceived impact on parental knowledge, attitudes, and behavior change: IFDC, Quantum Caring for Parents, and Peekaboo ICU Preemie (see Multimedia Appendix 1). Interestingly, Peekaboo ICU Preemie was not ranked among the highest in overall quality, but based on expert evaluation, the information within this app was found to likely enhance parents learning and feelings to support their NICU experience, despite receiving lower scores in the MARS core subscales. Unsurprisingly, a statistically significant difference was found between overall quality scores and subjective quality scores, with subjective quality ranking lower on average. This finding is expected as in comparison with the overall quality criteria (ie, the 4 core subscales), the subjective quality items are particularly broad. For example, 4 questions ask for the reviewers’ opinion on star rating, recommending the app, anticipated time using the app, and paying for the app. Those receiving a good score in the aesthetics subscale scored highest overall, suggesting these apps may have been developed with greater attention or to be in line with a fundamental theory of design in which *attractive things work better* [50]. The design of web-based resources, both websites or apps, should be a pleasurable experience for users and stimulate an overall positive response when interacting with the resource [51]. Design experts have outlined certain harsh conditions that produce a negative response often apparent in the NICU environment, including bright lights, loud abrupt noises, bodies that appear abnormal, etc [50]. Thus, it is important for these apps to use design techniques to enhance aesthetics as to not add to an already stressful experience in the NICU.

### Usability and Credibility

The information within many of the apps was found to be understandable and actionable by the general population as they provided content that used lay language or visual cues to identify important points. Interestingly, subscores related to quality were the most inconsistent across tools. Our subanalyses showed that the Trust It or Trash It tool was not correlated with MARS overall or subjective quality scores. On the basis of expert review using the MARS tool, apps received acceptable to good scores for the information quality subscale with most scoring high on item 15 (ie, *is the app content correct, well written, and relevant to the goal/topic of the app*) [39]. In contrast, when the same apps were measured using the Trust It or Trash It tool, the credibility of most apps was found to be questionable. There are several possible reasons for this difference. One reason may be that The Trust it or Trash Tool consists of 6 items, specific to quality, whereas the MARS tool is a single item. Additionally, the MARS tool does not include important questions related to credibility, such as identification of sources. Given the limitations of the MARS tool, a supplementary assessment of credibility should be considered. However, it is important to emphasize that although it is crucial to use criteria to assess credibility, current mobile apps may not weigh the criteria as strongly during development; that is, app developers may use evidence-based information in the development of content, yet not provide end users with where they obtained that information. Thus, it is possible the content may not necessarily be untrustworthy but simply does not provide a source and therefore, received a negative score using the Trust It or Trash It tool. However, this lack of provision of sources to parents would not be captured when solely evaluating using the MARS tool, resulting in the disconnect between these 2 scales. Parents are often limited in their ability to assess the reliability of health content, and mHealth apps that contain educational information should be held responsible to disclose important aspects related to credibility including original sources, author names, and bibliographies. Although not a focus of this review, it is important to note that 1 limitation of this review relates to the limitations of the tools currently available to evaluate the strength of health-related apps. The development of novel tools appears to be warranted.

### Secondary Objective

Relative to our secondary objective, item 19 of the MARS tool determines to what degree the app has been trialed and is considered to be evidence based. At the time of evaluation, only 1 app had a peer-reviewed publication associated with it (MyPreemie App); however, the identified study provided a description of the app’s content and features with no indication of empirical testing [42]. There is a published abstract discussing MyChildren’s app; however, there is no mention of the NICU specific content that was evaluated in this review [43]. Although identified after this review had been completed, the IFDC app was included in a recent publication regarding the developer’s larger parent education project. However again, it appears the app was not tested; thus, although meeting our secondary objective, it would not impact the MARS score [39]. The low quantity of peer-reviewed literature is not unique to our review. Creber et al in 2016 found only 3 publications out of 34 identified apps related to cardiovascular disease management [29], whereas Owens et al’s review (2018) of apps regarding chronic lung disease [33]and Tinschert et al on asthma-related apps found no publications [28]. The lack of existing peer-reviewed literature may put the apps identified in this review at a disadvantage as health care providers are less likely to recommend use of apps to patients and families if there is no evidence supporting their benefit. Thus, it is important for mHealth apps to provide an assessment of app quality through effectiveness studies and peer-reviewed publications [29].

### Limitations

Despite following a rigorous systematic approach with expert reviewers, synthesizing and evaluating mobile apps is still a relatively new concept and thus, there are some limitations to address. We did not include reviewers from outside the country and because of this, only apps accessible in Canada were included. Manually gathering apps during the initial search significantly prolonged this stage of the review. However, this has been resolved for future reviews as 1 author (MS) has since developed a method to retrieve all app data relevant for the search stage. Reviewers with different devices and operating system versions experienced differences in functionality and app store description information; however, this was resolved through discussion and briefly reviewing the apps again with each device to come to a consensus. This could be avoided in future reviews by using devices that are strictly for research purposes (ie, not the reviewer’s personal mobile phone). Due to the fact that we wanted to get an overview of all apps currently available to NICU parents, we not only included apps that were predominantly for monitoring and tracking data but also included other components such as a diary or a section to prompt questions to ask health care professionals. However, it was difficult to assess these apps using the PEMAT-AV as there was little health information within these apps. Moreover, we found that the PEMAT-AV may not be adequate to evaluate health literacy of mobile apps as the evaluation criteria have not yet been adapted to the unique variances within a mobile app platform. In addition, although the MARS tool was developed using rigorous methods, we would argue that most items in the core subscales elicit subjective responses, which could limit the objectivity and replicability of the measurement. The possibility for complete replicability of this review is limited because of the nature of mobile apps and various fluid components, including app ranking within stores, differences in devices, and recent updates of apps. Similarly, the findings of this review should be interpreted with some caution as the evaluation is based on the app version at the time of assessment, and thus, all future versions may differ in overall quality, usability, and credibility.

### Conclusions

Despite the number of available apps for parents of infants in the NICU, this systematic review revealed that current mobile app resources vary in quality, usability, and credibility, with generally low scores. Additionally, peer-reviewed literature or empirical studies related to the identified apps are nearly nonexistent. Parents should be aware of the issues of quality and credibility identified in this review and be cautious when using an app for health information. This expert review was beneficial to provide a preliminary evaluation on the resources easily accessible by a parent’s mobile phone. Building on this, usability testing or content analysis of the top 5 apps could be warranted to further explore how parents interact with the apps and provide a thorough evaluation examining the impact of these resources on parent learning needs, parent engagement, and neonatal outcomes. In addition, further attention to the development of high quality, credible resources targeted to NICU parents is needed.

## Appendix

Multimedia Appendix 1 Complete version of Table 1. Description of apps and Table 5. Mobile App Rating Scale average scores per app.

## Abbreviations

IFDC: Integrated Family Delivered Care
MARS: Mobile Application Rating Scale
mHealth: mobile health
NICU: neonatal intensive care unit
PEMAT-AV: Patient Education Materials Assessment Tool for Audiovisual Materials
PRISMA: Preferred Reporting Items for Systematic Reviews and Meta-Analyses
