# Supplementary material for: Evaluation of Mobile Apps Targeted to Parents of Infants in the Neonatal Intensive Care Unit: Systematic App Review
Source: JMIR Mhealth Uhealth. 2019 Apr 15;7(4):e11620. doi: 10.2196/11620 (PMC6487340; doi:10.2196/11620)
Supplement: Multimedia Appendix 1 [file mhealth_v7i4e11620_app1.pdf]

Table 1. Description of apps

App Name & Operating System	Developer	Country	Category	App Rating	Cost (CAD)	Version	Language(s)	Ads/In-app cost
Babble Apple iOS	Midcentral District Health Board	New Zealand	Medical	Not enough ratings	Free	1.5	English	No
Baby Growth Tracker Android	St. Rose Dominican Hospital	United States	Medical	3 stars	Free	1.1	English	No
Connect2NICU Apple iOS	Connect2 NICU	Unknown	Lifestyle	Not enough ratings	Free	1.1	English, French, Spanish, Arabic, German, Chinese, Catalan, Czech, Danish, Dutch, Finnish, Greek, Hebrew, Hungarian, Indonesian, Italian, Japanese, Korean, Norwegian, Bokmal, Polish, Portuguese, Romanian, Russian, Swedish, Turkish	No
Gift of Life Apple iOS	Mark Hoewing	United States	Medical	Not enough ratings	Free	1	English, Italian, Portuguese, Russian	Yes (Ads)
IFDC Android	Propeller Apps	United Kingdom	Medical	Not enough ratings	Free	1	English	No
Life’s Little Love Apple iOS	AlexiaTek	Canada	Medical	Not enough ratings	Free	1.0.8	English	No
myChildren’s Android	Nationwide Children’s Hospital	United States	Medical	5 stars	Free	4.2.1.1459-b6334bd	English	No
MyPreemie App Apple iOS	Graham’s Foundation	United States	Medical	Not enough ratings	Free	1.13	English, Spanish	No

My Neonatal Journal<br>Apple iOS	Rancon	United Kingdom	Medical	Not enough ratings	Free	1.1.1	English	No
NICU Companion<br>Apple iOS	Indiana University	United States	Medical	Not enough ratings	Free	2	English, Spanish	No
NICU Parent<br>Apple iOS	PSD Apps	Greece	Medical	Not enough ratings	Free	1.6	English	Yes (Ads)
Our Journey in the NICU<br>Apple iOS	Phoenix Children's Hospital	United States	Medical	Not enough ratings	Free	1	English, Spanish	No
Pebbles of Hope<br>Apple iOS	Pebbles of Hope	United States	Medical	Not enough ratings	Free	2	English	No
Peekaboo ICU Preemie<br>Android	Jozo Radman	United States	Medical	4 stars	Free	0.0.5	English	No
Premature Baby Journal<br>Android	Life's Little Treasures Foundation	Australia	Medical	Not enough ratings	$2.96	1.1	English	No
Premature Birth<br>Android	Health Care Tips	Unknown	Medical	4 stars	Free	1	English	Yes (Ads)
Quantum Caring for Parents (QCP)- NICU<br>Apple iOS	Caring Essentials	United States	Education	Not enough ratings	Free	1.1	English	Yes (In-app purchases)
Tommy's- My Premature Baby<br>Apple iOS	Tommy's	United Kingdom	Medical	Not enough ratings	Free	1.0.5	English	No

Table 5. Mobile App Rating Scale average scores per app

App Name	Engagement	Functionality	Aesthetics	Information Quality	Overall Quality	Subjective Quality	Perceived Impact
Babble	3	5	4.3	4	4.08	3	2.5
Baby Growth Tracker	2.2	3.25	3	3	2.86	1.75	2.5
Connect2NICU	2.6	4	1.67	3.2	2.87	1.75	2.5
Gift of Life	2	2.75	2	2.75	2.38	1	1
IFDC	3.8	4.25	4	4.2	4.06	3.5	4.67
Life's Little Love	2	4	3.67	3.8	3.37	2.5	3
myChildren's	3.2	3.5	3.3	3.8	3.45	2.25	2.5
MyPreemie App	3.2	4.25	4.67	4.5	4.16	4.25	3.5
My Neonatal Journal	3	3.75	3.3	3	3.26	2	1.33
NICU Companion	3.4	5	4	4	4.1	2.5	3
NICU Parent	1	4.25	1.3	2.75	2.33	1	1.33
Our Journey in the NICU	2	4.5	3	4	3.38	2.25	3.17
Pebbles of Hope	2.2	3.25	2.67	3.4	2.88	2	2.33
Peekaboo ICU Preemie	3.2	4	3.67	4	3.72	3.25	5
Premature Baby Journal	2.4	3.5	3.3	4	3.3	3.75	2
Premature Birth	1.4	4	2	2.8	2.55	1.25	2.17
Quantum Caring for Parents (QCP)-NICU	4.6	3.75	4.67	4.2	4.31	4	4.67
Tommy's- My Premature Baby	3	3.75	3.3	4.25	3.58	2.5	2.33
